# Nuclear Receptors Are Differentially Expressed and Activated in KAIMRC1 Compared to MCF7 and MDA-MB231 Breast Cancer Cells

**DOI:** 10.3390/molecules24112028

**Published:** 2019-05-28

**Authors:** Atef Nehdi, Rizwan Ali, Alshaimaa Alhallaj, Hajar Alzahrani, Nosaibah Samman, Abdullah Mashhour, Omar Baz, Tlili Barhoumi, Bandar Alghanem, Abdullatif Khan, Lolwah Alriyees, Mohamed Boudjelal

**Affiliations:** 1 Medical Research Core Facility and Platforms, King Abdullah International Medical Research Center (KAIMRC)/King Saud bin Abdulaziz University for Health Sciences (KSAU-HS), P.O. Box 3660, Riyadh 11481, Saudi Arabia; nehdiat@ngha.med.sa (A.N.); aliri@ngha.med.sa (R.A.); alhallajal@ngha.med.sa (A.A.); alzahraniha6@ngha.med.sa (H.A.); sammanno@ngha.med.sa (N.S.); mashhourab@ngha.med.sa (A.M.); bazom@ngha.med.sa (O.B.); barhoumitl@ngha.med.sa (T.B.); GhanemBa@ngha.med.sa (B.A.); 2 Department of Pathology and Laboratory Medicine, National Guard Health Affairs (NGHA), P.O. Box 22490, Riyadh 11426, Saudi Arabia; khanab4@ngha.med.sa; 3 Department of Surgery, National Guard Health Affairs (NGHA), P.O. Box 22490, Riyadh 11426, Saudi Arabia; lu_md@yahoo.com

**Keywords:** nuclear receptors, breast cancer, primary cells, drug discovery screening, estrogen receptor

## Abstract

We recently established a KAIMRC1 cell line that has unique features compared to the known breast cancer cell lines, MCF7 and MDA-MB231. To characterize it further, we investigated the expression profile of nuclear receptors and their respective co-factors in these cell lines. We confirm that in contrast to the triple negative cell line MDA-MB231, the MCF7 and KAIMRC1 are estrogen receptor alpha (ERa) and progesterone receptor alpha (PRa) positive, with significant lower expression of these receptors in KAIMRC1. KAIMRC1 cell is a vitamin D receptor (VDR) negative and V-ErbA-Related Protein 2 (EAR2) positive in contrast to MCF7 and MDA-MB231. Remarkably, the histone deacetylases (HDACs) are highly expressed in KAIRMC1 with HDAC6 and HDAC 7 are exclusively expressed in KAIMRC1 while thyroid hormone receptor-associated protein 80 (TRAP80), telomeric DNA binding protein 1 (TBP1) and TGF-beta receptor interacting protein (TRIP1) are absent in KAIMRC1 but present in MCF7 and MDA-MB231. In a luciferase reporter assay, the ERa coexpression is needed for estrogen receptor element (ERE)-luciferase activation by estradiol in KAIMRC1 but not in MCF7. The co-expression of exogenous Liver X receptor alpha (LXRa)/retinoid X receptor alpha (RXRa) are necessary for LXR responsive element (LXRE) activation by the GW3696 in the three cell lines. However, the activity of peroxisome proliferator-activated receptor response element (PPARE)-tk-luciferase reporter increased when peroxisome proliferator-activated receptors alpha (PPARa)/RXRa were coexpressed but the addition of PPARa agonist (GW7647) did not stimulate further the reporter. The signal of the PPARE reporter increased in a dose-dependent manner with rosiglitazone (PPARg agonist) in KAIMRC1, MCF7, and MDA-MB231 when the proliferator-activated receptors gamma (PPARg)/RXRa receptors were cotransfected. Retinoic acid-induced activation of retinoic acid receptor response element (RARE)-tk-luciferase is dependent on exogenous expression of retinoic acid receptor alpha (RARa)/RXRa heterodimer in MDA-MB 231 but not in MCF7 and KAIMRC1 cell lines. In the three cell lines, Bexarotene-induced retinoid X receptor response element (RXRE)-luciferase reporter activation was induced only if the RXRa/LXRa heterodimer were co-expressed. The vitamin D receptor response element (VDRE)-luciferase reporter activity showed another distinct feature of KAIMRC1, where only co-expression of exogenous vitamin D receptor (VDR)/RXRa heterodimer was sufficient to reach the maximum rate of activation of VDRE reporter. In the proliferation assay, nuclear receptors ligands showed a distinct effect on KAIMRC1 compared to MCF7 and MDA-MB231. Growth inhibition effects of used ligands suggest that KAIMRC1 correlate more closely to MDA-MB231 than MCF7. Vitamin D3, rosiglitazone, novel RXR compound (RXRc) and PPARa compound (GW6471) have the most profound effects. In conclusion, we showed that nuclear receptors are differentially expressed, activated and also their ligand produced distinct effects in KAIMRC1 compared to MCF7 and MDA-MB231. This finding gives us confidence that KAIMRC1 has a unique biological phenotype.

## 1. Introduction

The MCF7 and MDA-MB231 cell lines have been used for many decades as in vitro models for breast cancer research. These two cell lines represent two distinct breast cancer cell types; the estrogen receptor positive and negative breast cancer. However, the search for better in vitro breast cancer model system is always a quest as MCF7 and MDA-MB231 and other existing breast cancer cells have limitations and do not represent all the heterogeneity that exists in breast cancer. The newly established KAIMRC1 breast cancer cell line positive for both estrogen receptor alpha (ERa) and progesterone receptor alpha (PRa) proteins represent an alternative model [1]. In cancer research, including breast cancer, nuclear receptors represent one of the best and attractive drug targets due to their involvement as key players in physiological processes. In fact one of the most widely used anti-breast cancer drug, tamoxifen, works through the inhibition of estrogen receptor alpha activity [2,3,4,5,6].

Nuclear receptors (NRs) represent one of the most important cellular transcription factors that regulate essential genes involved in different cell functions like differentiation, metabolism, detoxification, death and survival [7,8,9,10]. More than 300 NRs exist in vertebrates, nematodes and insects, with only 48 reported in humans. Some of these receptors are regulated by known endogenous ligands and some (the orphan receptors) by yet to be discovered ligands [11,12]. Structurally, the NRs consist of a constitutively active N-terminal A/B domain containing the activation function 1 (AF1), the DNA binding domain (DBD) with two zinc fingers for interaction with hormone response elements (HREs) in their target genes, a hinge domain (D) and the ligand binding domain (LBD)or the E/F domain. The LBD is composed of 11-13 α-helices surrounding a hydrophobic binding pocket with C-terminal ligand-dependent function 2 (AF-2), nuclear localization signals and interaction motifs for heat shock proteins, coregulators and other transcription factors [8,13]. NRs bind to DNA as heterodimers, homodimers, or monomers. The receptors for the steroid ligands like glucocorticoid, progesterone, estrogen, androgen and mineralocorticoids bind to palindromic response elements as homodimers. The retinoid, vitamin D, Liver X receptors, and peroxisome proliferator receptors (RAR, VDR, LXRs and PPARs) and many orphan receptors, bind to DNA as a heterodimer with retinoid-x-receptor (RXR). While few nuclear receptors like DAX1 and SHP lack the DBD and do not bind the DNA, few others like constitutive androstane receptor (CAR) are constitutively active in the absence of ligand [11].

Structure- function studies on the activation mechanism of NRs led to the “mousetrap” model in which the ligand is trapped due the electrostatic potential of the receptor with a subsequent conformational change preventing the ligand exit. The agonist ligands lead to conformational changes that stabilize the NR structure compared to unligated protein and give the accessibility of the AF-2 domain to accessory proteins to activate the transcription. It also reveals new surfaces on NRs to recruit specific transcriptional coactivators. The antagonist binding, however, leads to improper conformational changes in NRs that do not lead to the transcriptional complex activation [9,10,11,14].

In breast cancer, two major estrogen receptors are involved, the estrogen receptors alpha and beta encoded by two separate genes [15,16]. In vitro, the MCF7 cell has been used extensively as a good model for the estrogen receptor alpha positive breast tumors while the MDA-MB231 and MDA-MB-435 cell lines do not express estrogen receptors alpha and beta. They are being used as model in studying the estrogen receptor negative breast tumor. The function of ERb is not clear in breast cancer but it forms a heterodimer with ERa and induces distinct gene expression when compared to ERa or ERb homodimers alone [3,4,17]. The ERa is expressed in almost 70–80% of the breast cancers and represents one of the best drug targets for non-metastatic condition [5]. In the estrogen receptor negative breast tumor, the therapy does not consider anti-estrogen as the receptor is silenced by epigenetic modification [18].

The peroxisome proliferator activated receptors (PPARs) exist in three isoforms namely, PPARa, PPARg and PPARb/d, and bind distinct ligands of long-chain polyunsaturated fatty acids, eicosanoid derivatives, and oxidized lipids [19]. These receptors regulate transcription through hetero-dimerisation with RXRa and regulate multiple cellular functions that include lipid biosynthesis and glucose metabolism [20]. The PPARs are linked to breast cancer because they bind and regulate the fatty acid metabolism and pathways. Long-chain n-3 polyunsaturated fatty acids showed a good inhibitory effect on the growth and metastasis of mammary tumor [21,22]. Also rosiglitazone, a PPARg specific ligand, promotes growth arrest and apoptosis in MCF7 cells in vitro [23]. Unfortunately, rosiglitazone did not show a significant clinical response in patients with metastatic breast cancer [24]. However, the PPARa ligand, Wy14643 and clofibrate increased MDA-MB-231 and MCF-7 cell proliferation [25]. The ability of PPARs to influence the adipocyte cell differentiation make them therapeutic targets through the modulation of the breast tumor stromal cells [21]

Many studies have shown an inverse correlation between breast cancer and uptake of vitamin D and calcium [26]. Vitamin D_3_ (1,25-dihydroxyvitamin D_3_ [1,25(OH)_2_D_3_]), the active form of VitD exerts its cellular activity through vitamin D receptor (VDR) which works as a heterodimer with RXRa. The VDR and vitamin D 1-hydroxylase, the enzyme that generates 1,25(OH)_2_D_3_, are expressed in the normal mouse mammary gland and human breast [27] and1,25-dihydroxyvitamin D_3_ [1,25(OH)_2_D_3_] has been shown to inhibit the breast cancer cell growth [28].

The cellular effects of vitamin D3 including cell growth arrest depend on the presence of nuclear VDR. In animal model, the vitamin D3 inhibited the cell growth of mammary cells but not in VDR −/− mice [29]. The 1,24(OH)_2_D_3_ also inhibited the growth of MCF-7 xenografts in nude mice by 50% [30]. EB1089, another analogue of 1,25(OH)_2_D_3_ showed the ability to suppress the mammalian cell proliferation in vivo [31]. All of these data show that VDR and its ligand have anti-breast cancer activity.

The liver X receptor alpha and beta (LXRa and b) bind the oxysterol receptors and have been shown to reduce the cell proliferation in tumor models [32]. Number of mechanisms have been proposed to address how the LXRs inhibit the cell growth such as inhibition of the growth factor inducing the PI3K-Akt pathway [32]. Also LXRs can increase the expression of p53 and maintain the level of retinoblastoma (Rb) while it decreases the ERa, Skp2, cyclin A2, and cyclin D1 expression [33,34].

Retinoids like vitamin A derivatives play an important role in cell differentiation and proliferation by modulating two retinoid nuclear receptor families namely, the retinoic acid receptors alpha, beta and gamma (RARs) and retinoid x receptor alpha, beta and gamma (RXRs). RARs bind to all-*trans*-retinoic acid (ATRA) and 9-*cis*-retinoic acid (9-*cis*-RA) while RXRs bind only to 9-*cis*-RA. RARs function only as heterodimers with RXRs, while RXRs can function both as heterodimers with the non-steroidal NRs like VDRs, PPARs, and LXRs and homodimers [35]. It is well documented that 9-*cis*-RA and its derivatives, bexarotene, inhibit the proliferation of many breast cancer cells and the all-*trans*-retinoic acid induce cell arrest [35].

9-*cis*-RA has been shown to suppress mammary tumor development in ER alpha negative breast tumor animal model and C3(1)-simian virus 40 large T-antigen mouse model [36]. However in human 9-*cis*-RA increase triglyceride with liver and skin toxicity and failed the Phase I clinical trial [37].

In parallel many biotech and pharma companies have developed better RAR synthetic compounds to be used as antiproliferatives both in ER alpha negative and positive tumor models. Many of them showed good in vitro efficacy but failed in clinical trials due to toxicity [38].

RXR selective ligands were also developed and showed good antiproliferative activity in vitro and in vivo in breast cancer tumor models. These compounds termed “rexinoids”were effective in the regression of the breast tumor developed in the mammary carcinoma in N-Nitroso-N-methylurea-treated rats (NMU model) [39]. The LGD1069 named bexarotene, a specific ligand for RXRa, suppressed effectively both ER-positive and ER-negative tumor development in NMU tumor model [40]. The bexarotene is being marketed as anti-ageing and anti-skin inflammation drug. The ability of RXRs to form heterodimer with other non-steroid nuclear receptor opens door for combination therapy with rexinoid and other nuclear receptor ligands like PPARs, VDR ligand and retinoids [41]. Novel rexinoids have been reported in literature and showed similar effect as Bexarotene [42]. We were also able to synthesize one rexinoid named RXRc and was tested in this study.

In this study, we assessed the expression and function of ERa, RARa, RXRa, VDR, LXRa, PPARa and g in KAIMRC1 cell in comparison to MCF7 and MDA-MB231. Our study demonstrate that the function of these receptors is distinct between these cell lines suggesting that KAIMRC1 represents another potential breast cancer cellular model that could be used to better understand the mechanism governing this disease and possibly allowing to discover novel anti-breast cancer drugs and biomarkers.

## 2. Results

The search for better anti-breast cancer drugs, particularly for the estrogen receptor alpha negative breast tumor, is a major focus of many researchers in academia and the pharmaceutical industry. In this regard non-steroidal nuclear receptors that include RARs, RXRs, VDR, LXRs and PPARs represent an alternative approach. In fact bexarotene, an RXR alpha ligand marketed for psoriasis was developed originally as an anti-breast cancer drug [39,40]. Moreover the latest understanding of the breast cancer biology is leading to new therapeutic approaches based on re-differentiation of breast cancer cells. Recently, it has been shown that induction of re-differentiation in breast cancer cells in a mice model, using PPAR gamma ligand, make them more sensitive to chemotherapy and kinase inhibitor-based therapy [43,44]. Many attempts failed to reproduce these results in vitro using the commonly used cell line models. Hence the necessity of new cellular system models able to mimic the behavior of primary cancer cells in tumor tissue is mostly needed. Breast cancer studies have used MCF7 and MDA-MB231 as cellular models for estrogen receptor positive and negative breast tumor respectively [26,27]. In this regard, our newly established and naturally immortalized breast cancer cell line (KAIMRC1) provides an alternative model for breast cancer research. To characterize KAIMRC1 further and enable its use as a cellular model for anti-breast cancer drug discovery, we investigated the function of multiple nuclear receptors in this cell in comparison to MCF7 and MDA-MB231 cells.

### 2.1. Nuclear Receptors and their Cofactor Expression in KAIMRC1 Compared to MCF7 and MDA-MB231

**Figure 1:**
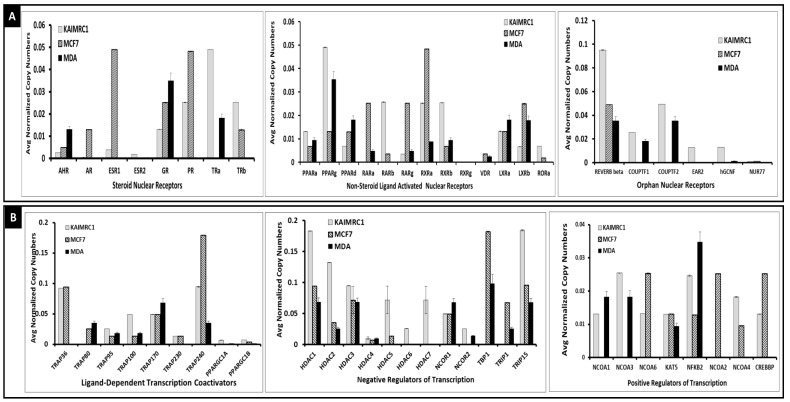
Nuclear receptors and their cofactors expression: The expression of the nuclear receptors (**A**) and their cofactors (**B**) were assessed in KAIMRC1, MCF7 and MDA-231 cells using the Qiagen RT² Profiler™ PCR Array Human Nuclear Receptors and Coregulators. The expression of steroid-thyroid, non-steroid/thyroid and orphan nuclear receptors as well as ligand activated cofactors, positive and negative regulators of transcription are plotted as an average of three independent experiments.

To study the functionality of nuclear receptors in KAIMRC1 cell, we first assessed their expression using the Qiagen Nuclear and coregulator RT^2^ Profiler PCR Array. The array profiles the expression of 84 genes encoding nuclear receptors and their coregulators. The array includes receptors for thyroid and steroid hormones, receptors for retinoids and vitamin D, as well as orphan receptors. Coactivators and corepressors of nuclear receptor activity are also included. As shown in Figure 1A, KAIMRC1 expresses lower levels of estrogen receptor alpha and progesterone receptor alpha compared to MCF7. Interestingly, KAIMRC1 also express low level of ERb that is absent in MCF7 under our conditions. Contrarily, KAIMRC1 expresses higher level of thyroid receptor alpha (TRa) than MDA-MB231 that is absent in MCF7 and higher level of thyroid receptor beta (TRb) than MCF7.

As for the non-steroid ligand-activated nuclear receptors, KAIMRC1 expresses different level of PPARs, RARs, RXRs and LXRa when compared to MCF7 and MDA-MB231. Interestingly, expression of VDR is absent in KAIMRC1 but present in MCF7 and MDA-MB231. KAIMRC1 expresses higher amount of REVERB alpha compared to MCF7 and MDA-MB231 and equal amount of COUPTF2 and 4 to MDA-MB231 but it is the only cell that express EAR2.

The expression of the nuclear receptor cofactors is summarized in Figure 1B under three categories: (i) Ligand-dependent transcription coactivators, (ii) negative and (iii) positive regulators of transcription. As shown in the Figure 1B, the expression of these cofactors is distinct in KAIMRC1 compared to MCF7 and MDA-MB231 cells. Interestingly, the histone deacetylases (HDACs) are highly expressed in KAIRMC1. HDAC6 and HDAC 7 are expressed exclusively in KAIMRC1 while no expression of TRAP80, TBP1 and TRIP1 was detected in these cells. The positive regulators of transcription, NCOA1 and NCOA3 are expressed only in KAIMRC1 and MDA-MB231 while NCOA6, NCOA4 and CREBBP are expressed in KAIMRC1 and MCF7 and absent in MDA-MB231. This data show that KAIMRC1 has a distinctive nuclear receptor and cofactors expression profile.

### 2.2. Transcriptional Activities of Nuclear Receptors in KAIMRC1, MCF7 and MDA-MB231 Cells

**Figure 2:**
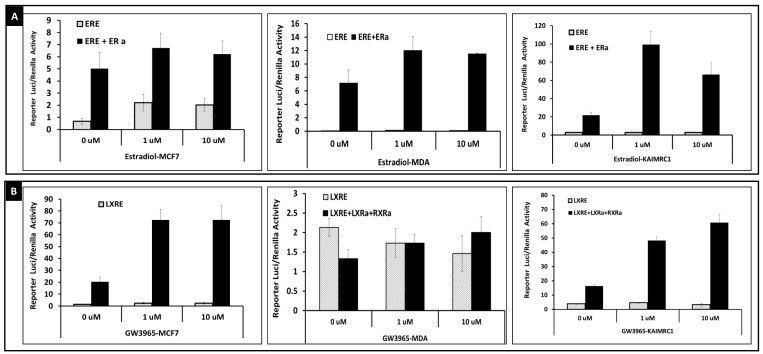

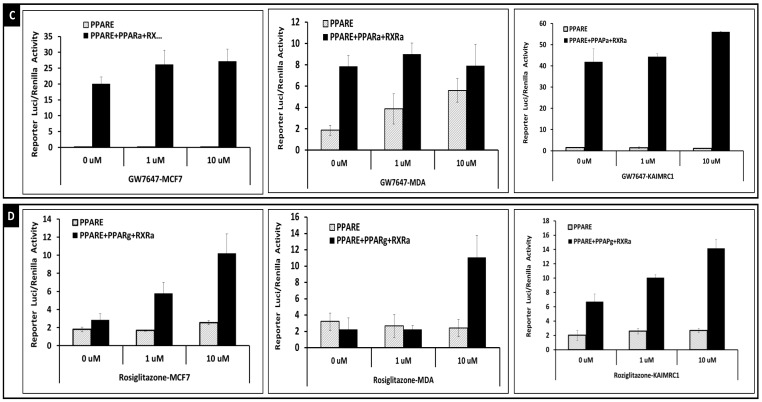

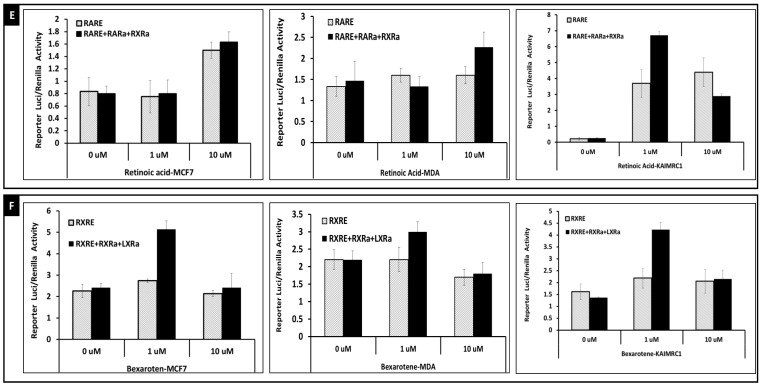

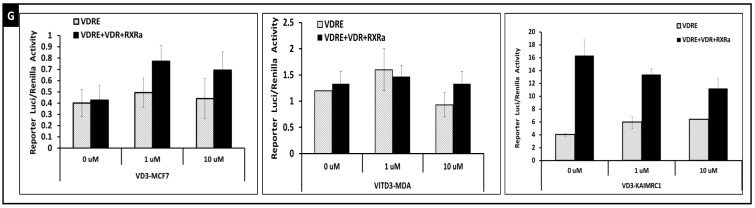
Nuclear receptors activity in KAMRC1, MCF7 and MDA-231: KAIMC1, MCF7 and MDA-231 cells were transfected with the following plasmids (**A**) ERE-3-Luciferase (pXP-ERE-3-luc) and pSG5-HEG0 (ERa) and treated with estradiol (**B**) LXRE-Luficerase reporter and pcDNA 3.2 LXR alpha) (**C**) PPARE(3)-tk-Luciferase plasmid, pcDNA3.1-Human PPARa, pcDNA3.1-hRXR alpha and treated with PPARalpha specific ligand GW7647 (**D**) PPARE(3)-tk-Luciferase plasmid, pSG5-hPPARgamma, pcDNA3.1-hRXR alpha and treated with PPARgamma ligand rosiglitazone (**E**) RARE-tk-luciferase, pSG5-hRARalpha, pcDNA3.1-hRXR alpha and treated with retinoic acid (**F**) RXRE (D1)-tk-luciferase, pcDNA3.1-hRXR, pcDNA 3.2 LXR alpha and treated with Bexarotene or RXR compound (RXRc) (**G**) VDRE-Luciferase plasmid, pSG5-hVDR, pcDNA3.1-hRXR and treated with Vitamin D3. 0.5 μg of pRL-CMV renilla was included in all the transfection, and compounds treatment were at 0, 1 and 10 μM. The reporter activity was calculated based luciferase activity/renilla activity.

We employed the luciferase reporter assay to assess the transcriptional activity of a number of nuclear receptors representing different classes. KAIMRC1, MCF7 and MDA-MB231 were transfected with different reporter plasmids where the luciferase-gene expression is driven by different nuclear receptor-sensitive response element. Reporter vectors were transfected alone or together with the corresponding activating nuclear receptor. Except for estrogen receptor alpha assay, all the nuclear receptors tested in this study, LXRa, PPARa, PPARg, RARa and VDR, were co-transfected with RXRa receptor as they are known to function as heterodimer with this receptor [42]. The activity of the reporter was assessed using the Dual-Glo luciferase assay kit. Our results showed that the estrogen receptor reporter (ERE-3-luficiferase) is constitutively active in MCF7 and its activity increases with estradiol treatment. In KAIMRC1 and MDA-MB231 cells, the ERE-3-luciferase reporter is induced by estradiol only if the estrogen receptor alpha is co- expressed (Figure 2A) that is in agreement with the previously published data for MDA-MB231 cells [45]. Unlike the MDA-MB 231, the KAIMRC1 and MCF7 express different level of estrogen receptor alpha. However, expression of exogenous estrogen receptor alpha is needed for ERE-3-luciferase reporter induction in KAIMRC1 but not in MCF7. This result indirectly indicates that the endogenous estrogen receptor alpha in KAIMRC1 is present but not active.

Figure 2B, summarize the transcriptional activity of LXRa and shows that the activation of LXRE-tk-luciferase reporter is dependent on the expression of exogenous LXRa. Co-expression of exogenous LXRa/RXRa increases the transcriptional activity of the reporter in all three cell lines. The reporter activity increased in dose dependent manner when cells were treated with GW3965, LXRa specific ligand.

The transcription activity of PPARa is shown in Figure 2C. In KAIMRC1 and MCF7 cells, the activation of PPARE(3)-tk-Luciferase reporter is dependent on the co-expression of exogeneous PPARa/RXRa, while in MDA-MB231, the reporter seems to be constitutively active, and it is responsive to the PPAR alpha ligand, GW7647. Co-expression of exogeneous PPARa/RXRa increased the reporter activity in all three cell lines. GW7647 treatment of cell overexpressing PPARa/RXRa did not induce any further the stimulation. The three cell lines showed a similar basal transcriptional activity of PPARg. Moreover, and in the absence of exogenous expression of PPARg/RXRa, all three cell lines were insensitive to the PPARE ligand, Rosiglitazone. Co-expression of exogenous PPARg/RXRa induces PPARE reporter further and the activation was boosted when cells were treated with the PPARg specific ligand, rosiglitazone. KAIMRC1 and MCF7 showed similar and higher sensitivity to rosiglitazone than MDA-MB231 cells. Based on these results, KAIMRC1 showed a closer behavior to MCF7 cell line than MDA-MB231.

We studied also the activity of retinoic acid receptor alpha (RARa) and retinoid x receptor alpha (RXRa). RARa was co-expressed with its reporter gene RARE-tk-luciferase and its heterodimer partner RXRa. As shown in Figure 2E, co-expression of exogenous RARa/RXRa, is not needed for retinoic acid-induced activation of RARE-tk-luciferase reporter in MCF7 and KAIMRC1 cell lines. MDA-MB231 showed the lowest sensitivity to retinoic acid stimulation even in presence of exogenous RARa/RXRa. Our data for MCF7 and MDA-MB231 is in agreement with previous published results [46].

Co-expression of RARa/RXRa heterodimer did not induce activation of RARE-tk-luciferase reporter in MCF7 and MDA-MB231 cells further in the presence of 1 uM retinoic acid but it with 10 uM. In contrast, in KAIMRC1, 1 uM retinoic acid treatment stimulated better the reporter when exogeneous RARa/RXRa were co-expressed but the luciferase signal was quenched when the cells were treated with 10 uM. This could be due to squelching effect. Again this data show that KAIMRC1 is distinct from MCF7 and MDA-MB231 in responding to retinoic acid stimulation for RARE-reporter in the presence of RARa/RXRA. As shown in Figure 2F, we tested the activity of retinoid x receptor by transfecting RXRE-reporter plasmid alone or in the presence of RXRa/LXRa. In this experiment, we also cotransfected RXRE-luciferase reporter with RXRa alone or together with RARa, PPARa and PPARg but the stimulation was not as high as compared to cotransfecting RXRa/LXRa (data not shown). In this assay, we tested the effect of the RXRalpha specific compound, bexarotene on reporter induction. We found that treatment with bexarotene had no effect in the absence of expression of exogenous RXRa/LXRa. When RXRa/LXRa were co-expressed, the RXRE-reporter increased similarly in the three cell lines when treated with bexarotene.

Lastly we tested the activity of vitamin D receptor using a VDRE-luciferase reporter alone or co-expressed with VDR and RXRa. As shown in Figure 2E, the VDRE reporter activity is not activated in MDA-MB231 even in co-expression of VDR/RXRa. In MCF7, co-expressing VDRE-luciferase reporter and VDR/RXRa, the luciferase activity increased when cells were stimulated with 1 uM and 10 uM vitamin D3. This data is in agreement with what has been published in [47]. In KAIMRC1, the expression of VDR/RXRa stimulated further the reporter even in the absence of vitamin D3. Vitamin D3 stimulation induces a slight a squelching effect. Again this data show that VDR activity in KAIMRC1 is distinct from that in MCF7 and MDA-MB231 cells

### 2.3. Effect of Nuclear Receptor Ligands on KAIMRC1 Proliferation Compared to MCF7 and MDA231 Cells

**Figure 3:**
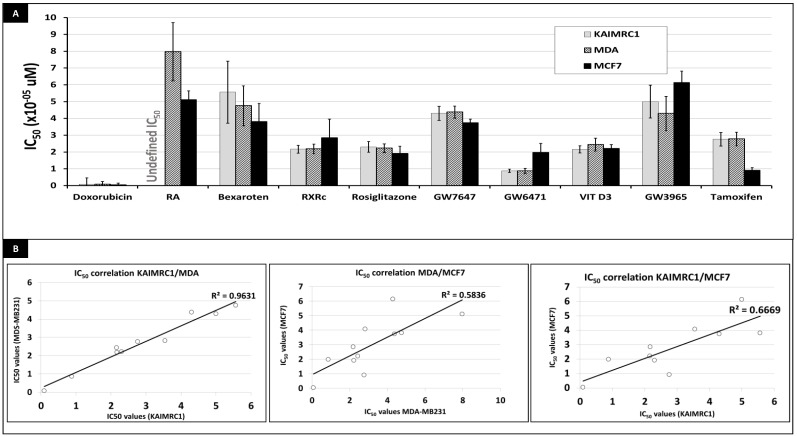
Cytotoxic effect of nuclear-receptor ligands (NRLs) on MDA-MB231, MCF7 and KAIMRC1 cell lines. IC_50_ values, showing the growth inhibitory effect of the different nuclear receptors on MDA-MB231, MCF7 and KAIMRC1 cell lines are represented in (**A**). IC_50_ values were used to make a comparative analysis of the sensitivity of the three cell lines to NRL-cytotoxicity. Effect-correlations are represented in (**B**).

The nuclear receptors represent one of the best target class to discover and develop new anti-cancer therapies. In this regard, we evaluated the effect of selected non-steroidal nuclear receptors on the proliferation of KAIMRC1, MCF7 and MDA-MB231 cells using the luminescence-based assay Cell-Titre Glo. The three cell lines were grown in complete media containing 10% charcoal stripped FBS and nine nuclear receptor compounds were added in a dose response manner starting at 100 uM as the highest concentration. Doxorobucin was used as internal control. After 48 h, the cell death was measured using TitreGlo as described in the Materials and Methods section. Figure 3A summarizes the cytotoxic/anti-proliferative effects (IC_50_) of the nine nuclear receptor-ligands tested. As expected, the positive control compound (doxorubicin) showed the highest cytotoxicity in all three cell lines. MCF7 showed the highest sensitivity to tamoxifen compared to KAIMRC1 and MDA-MB231. GW6471, PPARa antagonist, however, killed KAIMRC1 and MDA-MB231 at lower IC50, 0.8 and 1.9 respectively compared to MCF7.

Contrary to MCF7 and MDA-MB231, retinoic acid, a retinoid receptors ligand, did not kill KAIMRC1 even at very high concentration of 100 uM. Remarkably vitamin D3 (VitD3), rosiglitazone and RXRc killed all the three cells almost with the same IC_50_ (ranging from 1.9 to 2.4 uM). RXRc, synthesized in house is a ligand for retinoid X receptor alpha and is more potent that bexarotene in killing the three cell lines. To have a good comparison on the effect of the nine nuclear receptor ligands on the three cell lines, we performed a pairwise correlation test of the generated IC_50_. As shown in Figure 3B–D, KAIMRC1 IC_50s_ showed a high correlation score (R^2^ of 0.931) than their counterparts in MDA-MB231 cells. Correlation scores between MCF7/MDA-MB231 and KAIMRC1/MCF7 were (R^2^ of 0. 5836) and (R^2^ of 0. 6669), respectively. The full curves of are shown in Supplementary Figure S1. 

In conclusion and based on the sensitivity to nuclear receptor ligands anti-proliferation effect, KAIMRC1 seems to be more closely related to MDA-MB231 rather than to MCF7 cells.

## 3. Discussion

The search for novel cellular systems to discover new anti-cancer drugs is one of the challenges in biomedical research. In breast cancer research, scientists have extensively used the breast cancer cell lines MFC7 and MDA-MB231. MDA-MB231 is commonly used as a model of triple-negative breast cancer, while MCF-7 cells are used ubiquitously in ER-positive breast cancer studies [3,4,17]. The majority of the discoveries using these cell lines did not translate into marketed drugs due to poor efficacy in subsequent clinical trials. Our newly established breast cancer cell line, KAIMRC1 represents an attractive and alternative cellular model to be used for drug discovery as it is originated from a non-Caucasian patient and has a distinct cellular phenotype [1]. KAIMRC1 shares some phenotypes both with MCF7 and MDA-MB231 but has many unique features. Like to MCF, KAIMRC1 is positive for estrogen and progesterone receptors and negative for HER2. To explore its use in drug discovery, especially for nuclear receptor modulators, we first explored the expression and function of number of nuclear receptors and their cofactors in KAIMRC1 in comparison to MCF7 and MDA-MB231 cells. Nuclear receptors are transcription factors that are grouped into two subfamilies, the steroid hormones that include estrogen receptors (ERs), glucocorticoid receptors, progesterone receptors (PRs), androgen receptors (ARs) and the thyroid hormone (TRs). The non-steroid nuclear receptors subfamily comprises vitamin D receptor (VDR), retinoic acid receptors (RARs), retinoid X receptors (RXRs), the peroxisome proliferator-activator receptors (PPARs), Liver X receptor, Farnesoid X receptor (FXR) and the orphan nuclear receptor for which the endogenous ligand has not been identified [48,49].

We explored the expression of nuclear receptors using QPCR. As shown in Figure 1A, at the mRNA level, KAIMRC1 is still expressing estrogen receptor alpha but at a relatively lower level than MCF7. Progesterone receptor alpha is also expressed in KAIMRC1 at a higher level than the estrogen receptor alpha. Based on this data KAIMRC1 is still considered as an ER+/PR+ cell line, but these two receptors are expressed with a lower level if compared to MCF7. KAIMRC1 cells also express ERb that is absent in MCF7 and MDA-MB231 as shown in Figure 1A. Expression of ERb receptor is a distinctive feature of KAIMRC1 that makes from this cell a good model to explore the biology of ERb in breast cancer. The role of ERb in breast cancer is not yet clear, but it was shown that it hetero-dimerizes with ERb to induce specific genes expression [3,4,17]. Recently Song et al. [50] found that ERb expression was lower in tumor tissue than adjacent normal tissue of breast cancer patients. They showed also that over expression of mitochondrial ERb increases ATP production in triple negative breast cancer cells and normal breast cell (MCF10-A). Their results indicate that up-regulation of mitochondrial ERb in breast cancer cells ensures proper mitochondrial transcription and production of ATP. Expression of higher level of thyroid receptor alpha (TRa) and beta (TRb) represents another unique feature of KAIMRC1 cells. The role of thyroid hormone and its receptors in breast cancer etiology and progression is not well studied. A statistically significant association between high TRa expression and breast cancer has been reported [51,52]. The expression of other non-steroidal nuclear receptors PPARs, RARs, RXRs and LXRa in KAIMRC1 is distinct compared to MCF7 and MDA-MB231 as shown in Figure 1A. Interestingly, KAIMRC1 was found to be VDR negative, while both MCF7 and MDA-MB231 express this receptor. Moreover, KAIMRC1 is the only cell line that expresses the orphan nuclear receptor EAR2 and a higher amount of REVERB alpha in comparison to MCF7 and MDA-MB231. Most of these nuclear receptors are involved directly or indirectly in breast cancer [53]. All of these distinctive features make KAIMRC1 a new model for studying new aspects of breast cancer biology.

Nuclear receptors function as transcription factors to regulate the gene-expression through the interaction with other coactivators and corepressors [54]. Nuclear receptor coregulators such as steroid receptor coactivator-1 (SRC-1), transcription intermediary factor 2 (TIF2) and the NR corepressor (N-CoR) have been shown to play a role in breast cancer tumor progression [55]. SRC-3 is the well-studied cofactor in the breast cancer. It recruits histone acetyl transferases and methyltransferases for chromatin remodeling to facilitate gene transcription [56]. SRC-3 is overexpressed in breast cancer and nowadays is used as a prognostic marker, and a predictive marker of response to endocrine therapy [57].

As shown in the Figure 1B, the expression of number of these cofactors is distinct in KAIMRC1 compared to MCF7 and MDA-MB231 cells. Interestingly, the histone deacetylases (HDACs) are highly expressed in KAIRMC1 with HDAC6 and HDAC 7 expressed exclusively in KAIMRC1. Histone deacetylase inhibition constitutes an attractive therapeutic strategy for breast cancer [58]. Similarly to coactivators, some co-repressors of nuclear receptors have been shown to have altered expression in breast cancer. For example, the nuclear receptor corepressors (N-CoRs) have been shown to be downregulated in breast cancer and it is used is a marker of resistance acquisition to tamoxifen [59]. Low N-CoR expression is associated with significantly shorter relapse-free survival [60]. The positive regulators of transcription, NCOA1 and NCOA3 are expressed only in KAIMRC and MDA-MB231 while NCOA6, NCOA4 and CREBBP are expressed in KAIMRC and MCF7 and absent in MDA-MB231. This data show that KAIMRC1 has a distinct expression profile of nuclear receptors and their co-factors.

The nuclear receptors are important transcription factors and to assess the transcriptional activity in KAIMRC1, MDA-MB-231 and MCF7, we used luciferase reporter assays [45]. As detailed in the Results section, KAIMRC1 cells showed a distinct nuclear receptor reporter activity as compared to MCF7 and MDA-MB231 cells. Although KAIMRC1 is an ER alpha positive, no basal activity of ERE-luciferase reporter was detected even after stimulation with estradiol. Expression of exogenous estrogen receptor alpha was needed for reporter activation in KAIMRC1. This data indicates that the endogenous ER alpha in KAIMRC1 may not be susceptible to estradiol activation. This gives KAIMRC1 a unique phenotype to explore the biology of estrogen receptor alpha function taking into consideration that the ERa gene is not mutated as confirmed by exome sequencing of KAIMRC mRNA (data not shown). The response of LXRE-luciferase reporter is active in all the three cell lines and gets activated by the GW3965, LXR alpha specific ligand when exogenous LXRa and RXRa were coexpressed. The data is consistent with the previous studies on MCF and MDA-MB231 [61].

For PPARE-tk-luciferase reporter activity, MDA-MB-231 was the only cell line to be sensitive to the PPAR alpha specific ligand (GW7647) stimulation. MCF7 and KAIMRC1 responded to stimulation only when PPARa/RXRa were co-expressed with the reporter. In the three cell lines, co-expression of exogenous PPARa/RXRa was sufficient to activate the luciferase reporter in the absence of any stimulation. This indicates that the PPARa pathway is constitutively active in the three cell lines. To confirm that this effect is driven by PPARa, we used the PPARa antagonist (GW6471). Treatment of cells with this antagonist induced a dose-dependent decrease of reporter signal (data not shown).

The response of PPARE-tk-luciferase to rosiglitazone, a PPARg ligand, was found to be totally different to the effect observed with PPARa ligand. As shown in Figure 1, all three cell lines responded to rosiglitazone stimulation only when the PPARg/RXRa receptors were co-expressed. The signal increased in a dose-dependent manner in all three cell lines.

Heterodimerization and cross-talk between nuclear hormone receptors exist. For example, estrogen receptor alpha (ERa) physically binds to peroxisome proliferator-activated receptor gamma (PPARg) and inhibits its transcriptional activity. Also PPARg physically associates with VDR in human breast cancer cells. Overexpression of PPARg decreased 1α,25-dihydroxyvitamin D(3) (1,25D(3)) -mediated transcriptional activity of the vitamin D target gene, CYP24A1 by 49% and the activity of VDRE-luc, a vitamin D responsive reporter, by 75% in T47D human breast cancer cells [62,63].

In MDA-MB-231 cells, RXR was not associated with active transcription site in the presence of ligand. In addition the ligand-dependent activation of homo- or heterodimer RXR transactivation using RXR response element or RARE showed only minimal response to ligand treatment in MDA-MB231 cells. However in MCF7, these promoters were active and responsive to ligand activation [64,65]. Similarly in our assay in MDA-MB231 the retinoic acid alone did not activate the RARE-tk-luciferase in MDA-MB231 and was possible only after coexpression of RARa/RXRa herodimer. However in MCF7 and KAIMRC1, the promoter was activated even without coexpression of the RARa/RXRa heterodimer. However, for the RXRE-luciferase reporter, the signal was induced by bexarotene, RXR specific ligand, only if the RXRa/LXRa heterodimer was co-expressed. The induction was higher in MCF7 and KAIMRC1 compared to MDA-MB231. This data is aligned with what has been reported for MCF7 and MDA-MB231 cells [64,65] and indicates that KAIMRC1 cells resemble more withMCF7 than the MDA-MB231 cells.

The VDRE-luciferase reporter activity is another distinct feature of KAIMRC1. As shown in Figure 1, the reporter is not active in KAIMRC1 and co-expression of VDR/RXRa heterodimer and ligand addition does not increase the activity further. In contrast to MCF7, ligand treatment (vitamin D3) increased the reporter signal after VDR/RXRa co-expression. For MDA-MB231, the reporter was inactive even after vitamin D3 treatment and VDR/RXRa co-expression. First this data is in alignment of what has been published in [66]. Secondly it demonstrates that KAIMRC1 is distinct than MCF7 and MDA-MB231 cell for the VDR pathway.

In the proliferation assay, vitamin D3 showed a prominent effect on MFC7, MDA-MB231 and KAIMRC1 cells. Even though expression of VDR in mammary gland and breast tumors has been identified in the early 80s, the evidence for correlation between breast cancer sensitivity to vitamin D3 and the expression of VDR is still controversial. Certain studies showed that in breast cancer, the vitamin D receptor (VDR) acts as a master transcriptional regulator of many genes involved in autophagy [66,67]. In tamoxifen-resistant MCF7, expression of VDR re-sensitize cells to tamoxifen [68]. Model systems of carcinogenesis have provided evidence that both VDR expression and vitamin D3 action changes with transformation but the clinical data regarding vitamin D responsiveness of established tumors is limited and inconclusive [69]. These studies correlate well with the fact that we found VDR to be expressed in MCF7 and MDA-MB231 but absent in KAIMRC1, however the three cell lines are equally sensitive to vitamin D3 antiproliferative effect. In this regard, KAIMRC1 represent a unique model to better understand pathways triggered by vitamin D3 and inducing cell death in breast cancer.

Lastly, bexarotene and RXRc, ligands for RXRa, induced cell death in the three cell lines similar to rosiglitazone. However, RXRc and rosiglitazone were more potent, with IC_50_ values ranging from 19–28 uM respectively. Our data agrees with the studies showing that rosiglitazone induces apoptosis and authophagy in breast cancer cells [44]. The most interesting effect is the ability of GW6471, a PPAR alpha antagonist, to kill MCF7 and MDA-MB231 with an IC50 of 0.8 uM and kAIMRC1 with an IC_50_ of 1.9 19 uM. It has been shown that PPARa is active in breast cancer and its inhibition leads to cell death [25]. The general concept and understanding based on experimental work is that PPARs play an important role in breast cancer in an opposite manner. The ability of PPARs to influence the adipocyte cell differentiation makes it a potential therapeutic target by modulating the breast tumor stromal cells [21].

In conclusion, we showed that nuclear receptors are distinctly activated in KAIMRC1 compared to MCF7 and MDA-MB231. This finding gives us an advantage to use this cell line to screen for novel nuclear receptor modulators which could be used as future anti-breast cancer drugs.

## 4. Materials and Methods

### 4.1. Chemicals

All the compounds were purchased from Tocris Bioscience (Minneapolis, MN, USA). The compound RXRc reported in Yamada and Kakuta [42] was synthesized in house.

### 4.2. Plasmids

The following plasmids were obtained as a gift from Prof. Simak Ali, (Imperial College, London, UK), ERE-3-Luciferase (pXP-ERE-3-luc); pSG5-HEG0 (ERa), pSG5-hVDR, VDRE-Luciferase; PPARE(3)-tk-Luciferase, Human PPARa in pcDNA3.1, pSG5-hPPARgamma. The remaining plasmids (pcDNA3.1-hRXR alpha, pSG5-hRARalpha, pcDNA3.2 LXR alpha, RXRE (D1)-tk-luciferase, RARE-tk-luciferase, LXRE-tk-Luciferase reporter) were previously constructed. pRL-CMV Vector for renilla was purchased from Promega (Madison, WI, USA).

### 4.3. Cell Culture

Human breast cancer epithelial cell lines, MDA MB-231 (HTB26), MCF-7 (HTB-22) and KAIMRC1 (1) were maintained in Dulbecco’s Modified Eagle Medium (DMEM) supplemented with 10% fetal bovine serum (FBS), 50 units/ ml penicillin and 50 μg/mL streptomycin (Gibco, Gaithersburg, MD, USA), 2 mM L-glutamine (Gibco) at 37 °C in a humidified 5% CO_2_ atmosphere. All the proliferation and reporter assays were performed in assay media composed of phenol free DMEM containing 10% charcoal stripped FBS, 50 units/ ml penicillin and 50 μg/mL streptomycin (Gibco), 2 mM L-glutamine (Gibco).

### 4.4. Cell Transfection

0.5 × 10^6^ KAIMRC1, MCF7 or MDA-MB231 cells were seeded in each well of 6 wells plate in 2 mL complete media. Two hours later, the transfection mix was added that was prepared as follow: In 100 μL Opti-MEM I Reduced Serum Media (Thermo Fisher Scientific, Bartlesville, OK, USA), 2.5 ug reporter plasmid alone or with 0.5 ug of the corresponding nuclear receptors (as indicated in the figures) was added. 0.5 ug pRL-CMV Vector plasmid was cotranfected in all conditions for renilla measurement. Then 10 μL FuGENE^®^ 6 Transfection Reagent (Promega) were added to the transfection tube in drop wise. After 10 min incubation, the transfection mix was added to the cells, one tube of transfection mix (100 uL) was added to one well of cultured cells in 6 wells plate. After 24 h transfection, the media was switched to charcoal stripped phenol-free complete media for another 24 h prior seeding the cells in the compound plate.

### 4.5. Luciferase Reporter Assays

The 96-wells assay plate (white opaque) containing compounds were prepared by adding 1 μL of compound dissolved in DMSO per well in triplicates. The transfected cells cultured in 6 well plates were trypisinized and re-suspended in assay media (phenol-free complete DMEM media containing 10% charcoal stripped fetal bovine serum). Cells (10,000 cells in 100 μL per well) were added to the compound plate per well. After 24 h, media was replaced in each well with same fresh media and 1 µL of desired compound dissolved in DMSO or DMSO alone as control was added to each well to give the final concentration 0.1, 1 or 10 μM. The cells were then continued to be cultured for another 24 h prior assaying the luciferase activity using Dual-Glo^®^ Luciferase Assay System (Promega). One hundred μL of the Dual-Glo^®^ Luciferase buffer containing luciferase substrate were added to measure firefly luciferase (the promoter activity) and read on a Envision 2105 Multimode Plate Reader (PerkinElmer, American Fork, UT, USA). Then 100 μL of Stop-Glo buffer containing Stop-Glo substrate to measure renilla and plate were read again in the Perkin Elmer Envision system. The reporter induction was calculated by dividing the luciferease signal on the renilla signal.

### 4.6. Cell Proliferation Assay

5000 cells (KAIMRC1, MCF7 or MDA-MB231) in 100 µL assay media were seeded into 96-assay plate containing 1 μL of compounds (dissolved in DMSO) at desired concentration. The plates were incubated in 37 °C in a humidified 5% CO2 atmosphere for 48 h and then 100 μL CellTiter-Glo^®^ reagents (Promega,

San Luis Obispo, CA, US) and luminescence was measured using the PerkinElmer Envision instrument.

### 4.7. RNA Preparation and cDNA Synthesis

Total RNAs from KAIMRC1, MCF7 and MDA-MB231 cells were extracted using the PureLink RNA mini kit (ThermoFisher Scientific, Bartlesville, OK, USA). The isolated RNAs were quantified and quality checked using the Nanodrop 8000 8-well spectrophotometer (ThermoFisher, Bartlesville, OK, USA). 400 ng from each total RNA was used to prepare first-strand synthesis using the high capacity cDNA Reverse Transcription Kit (Applied Biosystems, Foster City, CA, USA) on PCR using the following parameters: 25 °C for 10mins, 37 °C for 120 min, 85 °C for 5mins and finally held at 4 °C. The expression of nuclear receptors and their cofactors were analyzed using RT2 Profiler PCR Arrays PAHS-056Y from Qiagen (Germantown, MD, USA). Glyceraldehye-3-phosphate dehydrogenase (GAPDH) expression was used as the internal control. All reactions were performed in a MicroAmp optical 96-well plate (Applied Biosystems) in a final volume of 10 μL per reaction.

The amplification was performed using the 7900 HT Fast Real-time PCR system (ThermoFisher, Bartlesville, OK, USA) using 1 μg cDNA and the following PCR cycling parameters: 95 °C for 10 min followed by 40 cycles of PCR reactions at 95 °C for 30 sec and 60 °C for 1 min. The relative expression levels of the genes assayed in the cell synchronization validation method experiment were calculated using the comparative threshold cycle Ct (ΔΔCt) method. Initially, the Ct value of each gene was normalized to the corresponding Ct value of GAPDH for the same sample to obtain the relative threshold cycle (ΔCt). Following this the ΔCt for each biological replicate was then exponentially transformed into ΔCt expression by calculating the value of 2 raised to the -ΔCt. Next, the average and standard deviation of the biological replicates were calculated followed by normalization to the average of the control samples for the same gene (ΔΔCt). Finally the ΔΔCt was calibrated from control samples and expressed as a relative fold-change. For relative mRNA analysis, raw Ct values were exported from ABI 7900 and imported into Microsoft Excel. Ct values were then used to calculate copy number for each well using historical/generic values from standard curves (intersection at 0 = 40, slope = –3.5) (data not shown). Copy numbers were then normalized to GAPDH and mRNA levels were expressed relative to control samples.

## Supplementary Materials

Supplementary Figure S1: Growth inhibition effect of nuclear-receptors ligands (NRLs) on MDA-MB231, MCF7 and KAIMRC1 cell lines.

## Abbreviations

Nuclear receptors (NRs), estrogen receptor (ER), retinoic acid receptor (RAR), retinoid x receptor (RXR), liver x receptors (LXRs) peroxisome proliferator receptors (PPARs), vitamin D receptor (VDR); abbreviations of all other nuclear receptors and their cofactors investigated in this study could be found through the following link: https://www.qiagen.com/fr/shop/pcr/primer-sets/rt2-profiler-pcr-arrays/?catno=PAHS-056Y#geneglobe.
