# Supplementary material for: Nuclear Receptors Are Differentially Expressed and Activated in KAIMRC1 Compared to MCF7 and MDA-MB231 Breast Cancer Cells
Source: Molecules. 2019 May 28;24(11):2028. doi: 10.3390/molecules24112028 (PMC6600534; doi:10.3390/molecules24112028)
Supplement: Supplementary file 1 [file molecules-24-02028-s001.pdf]

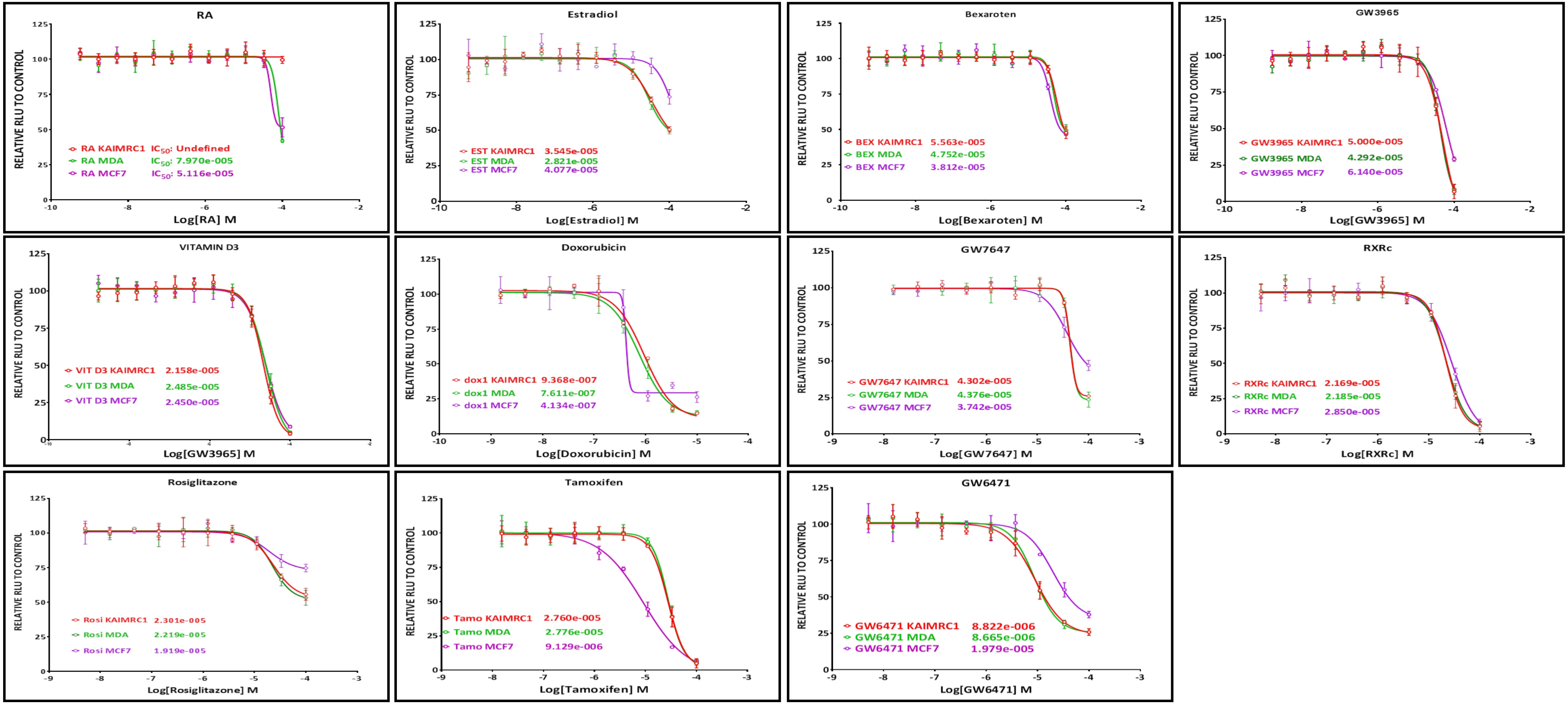
RA
RELATIVE RLU TO CONTROL
Log[RA] M
RA KAIMRC1 IC_{50}: Undefined
RA MDA IC_{50}: 7.970e-005
RA MCF7 IC_{50}: 5.116e-005
Estradiol
RELATIVE RLU TO CONTROL
Log[Estradiol] M
EST KAIMRC1 3.545e-005
EST MDA 2.821e-005
EST MCF7 4.077e-005
Bexaroten
RELATIVE RLU TO CONTROL
Log[Bexaroten] M
BEX KAIMRC1 5.563e-005
BEX MDA 4.752e-005
BEX MCF7 3.812e-005
GW3965
RELATIVE RLU TO CONTROL
Log[GW3965] M
GW3965 KAIMRC1 5.000e-005
GW3965 MDA 4.292e-005
GW3965 MCF7 6.140e-005
VITAMIN D3
RELATIVE RLU TO CONTROL
Log[GW3965] M
VIT D3 KAIMRC1 2.158e-005
VIT D3 MDA 2.485e-005
VIT D3 MCF7 2.450e-005
Doxorubicin
RELATIVE RLU TO CONTROL
Log[Doxorubicin] M
dox1 KAIMRC1 9.368e-007
dox1 MDA 7.611e-007
dox1 MCF7 4.134e-007
GW7647
RELATIVE RLU TO CONTROL
Log[GW7647] M
GW7647 KAIMRC1 4.302e-005
GW7647 MDA 4.376e-005
GW7647 MCF7 3.742e-005
RXRc
RELATIVE RLU TO CONTROL
Log[RXRc] M
RXRc KAIMRC1 2.169e-005
RXRc MDA 2.185e-005
RXRc MCF7 2.850e-005
Rosiglitazone
RELATIVE RLU TO CONTROL
Log[Rosiglitazone] M
Rosi KAIMRC1 2.301e-005
Rosi MDA 2.219e-005
Rosi MCF7 1.919e-005
Tamoxifen
RELATIVE RLU TO CONTROL
Log[Tamoxifen] M
Tamo KAIMRC1 2.760e-005
Tamo MDA 2.776e-005
Tamo MCF7 9.129e-006
GW6471
RELATIVE RLU TO CONTROL
Log[GW6471] M
GW6471 KAIMRC1 8.822e-006
GW6471 MDA 8.665e-006
GW6471 MCF7 1.979e-005

Supplementary Figure 1: Growth inhibition effect of nuclear-receptors ligands (NRLs) on MDA-MB231, MCF7 and KAIMRC1 cell lines. To determine their differential sensitivity to NRLs, the three cell lines MDA-MB231, MCF7 and KAIMRC1, were incubated for 48 with a gradient concentration of the different NRLs. Growth inhibition/cytotoxicity was assessed using the luminescence-based assay cell-Titer glo and represented (with IC_{50} values) as dose-response curves.
